# The use of social media inside and outside the classroom to enhance students’ engagement in EFL contexts

**DOI:** 10.3389/fpsyg.2022.1005313

**Published:** 2022-09-23

**Authors:** Hui Wang, Minqi Wang, Guang Li

**Affiliations:** Faculty of Education, Northeast Normal University, Changchun, China

**Keywords:** technological advances, social media, students’ engagement, EFL contexts, language learning

## Abstract

It could be claimed that without any doubt the Internet has revolutionized the educational system to a great extent. Even though some are still interested in traditional ways of teaching and learning and also face-to-face classes, technological advances, in particular, social media have changed the English as a foreign language (EFL) contexts in a way that they will not be compatible with any other methods that have long been utilized before. Despite the fact that some studies have been conducted in different learning contexts, to the researcher’s best knowledge, attention has not been focused on the importance of social media inside and outside the classroom on EFL students’ indulgence. Therefore, the aim of this review is to synthesize the findings of the previous studies to highlight the role of social media in EFL students’ engagement throughout class and outside the class in both face-to-face and online classes. To reach this goal, this review first discusses the impact of social media on engagement, and then engagement and its classifications are dealt with. Next, the relationship between using social media and students’ engagement is discussed. And finally, some implications are proposed.

## Introduction

It is undeniable that the internet and technological advances have paved the way that one can learn and teach a new language. As technology has developed and social media has attracted attention, teachers have been given more opportunities to utilize technology to enhance teaching a foreign language. In recent years, there has been a general positive inclination toward technology-based education since it is time-saving and much more interactive. On the other hand, even though autonomy is the ultimate aim in the educational system and good teachers strive hard to make their students independent in the process of learning, some students have no tendency to use this kind of tool because they have always wanted to be supported by their teachers and cannot just use these tools as an alternative to the traditional way of teaching in which teachers are highlighted. Regarding social media both inside and outside the class, it has been believed that both interaction and engagement can be enhanced through it (Al-Khalidi and Khouni, 2021). Moreover, students can create their own content at their preferable pace and it can be customized as well (Goel and Singh, 2016). In recent years, students’ engagement is highly impacted by Social media. Engagement, on the other hand, is perceived as an important part of the process of learning. It is divided into various categories: Emotional engagement, for instance, when one is highly passionate about the activity and feels less anxious and tired. The second category of engagement is cognitive involvement in learning strategies and self-use restrictions. Agent participation as another category of engagement is a conscious effort for the learning experience to be enriched (Hiver et al., 2021). Even though the significance of social media in the EFL contexts has been discussed in some studies before, just a few studies have considered the impact of social media on students’ engagement both inside and outside the class. To bridge the gap, this review study considers the impact of social media on engagement, then engagement and its categories are dealt with. Next, the relevance between utilizing social media and students’ engagement is discussed. And finally, some implications are put forward.

## Background

### The impact of social media on education

Studies have indicated to what extent it is significant to consider the role of social media in EFL students’ engagement. Researchers and educators have tried to find ways to cause EFL students to apply social media such as YouTube and Wikipedia in an interactive way. In an educational context, information technology has encouraged pedagogical practices through which practicable teaching and learning methods have been redefined. Social media platforms are a good example of these new technologies. They have been perceived by teachers as a force drive among teachers and learners. According to Kaplan and Haenlein (2010), social media is defined as “a group of Internet-based applications that build on the ideological and technological foundations of Web that allow the creation and exchange of user-generated content. It is a medium for social interaction as a super-set beyond social communication enabled by ubiquitously accessible and scalable communication techniques” (p. 63). The collaboration which is an important part of engagement is supported by social media, and important information sources are provided as a result of active involvement among learners by these platforms. As known, with the advent of social media, those EFL learners who received information passively have turned into those creating active content (Haque and Al Salem, 2019). Without any doubt, creativity is another paramount factor, considering engagement. The following terms, social networking and social media can be interchangeably utilized. However, social media is an umbrella term that helps convey messages. The first study on the use of social media in education has been conducted by Baird and Fisher (2005). Moreover, the significant advantages of social media platforms for learners were identified by them. Students these days have been brought up in a world where the Internet and social media play a pivotal role. Hence, their expectations and learning styles have changed in comparison with the past generation. In the traditional way of learning, no emphasis was placed on making students independent and content creators; they were supposed to receive information; therefore, creativity played no pivotal role in their learning process. These days, students access electronic libraries, different types of information and social networking tools (Elsayed, 2011).

Nowadays, students can create their own content whose pace can be chosen by themselves and it also may be customized so that it can be perceived as engagement outside the class (Goel and Singh, 2016). It is not just students that have been affected by social media, but teachers are also impacted because they use social media that are supposed to support students and be active members of this collaborative learning community. According to Dabbagh and Kitsantas (2012), higher education institutions in some countries still trust traditional ways of learning and teaching, they are course-based, for example despite the fact that the spread of social media and its positive applications can make a difference in the way of learning and teaching. Another study carried out by Al-Sharqi et al. (2015) showed how students are influenced by social media, and the students claimed that social media helped them understand how people think and made them able to connect with various communities and learned to be more open-minded. It was also believed that social media reduced their boredom and alleviated their monotony which tremendously affect the way they are engaged in activities both inside and outside the class. On the other hand, those who were exposed to social media reported distraction and unproductivity as drawbacks which again have a negative impact on students’ involvement. Group discussions, information access, resource sharing, and entertainment can be provided by social media and all of which are highly important for engaging the students. Furthermore, it was claimed that English proficiency could be enhanced by viewing and commenting on their friends’ posts on Facebook which can be considered as advantageous, meaning that not only students can be involved in the activities in the class, but they can also practice after class (Shih, 2011).

Currently, it is claimed that not only is knowledge found in individuals but it can also be found when interacting with others. Therefore, active involvement is supported through such interactions which is really crucial in the learning process of students (Hrastinski, 2009). In other words, interaction is a crucial part of the engagement. Skills should be developed to transfer information and to acquire knowledge with others that are provided both in the contexts which are face-to-face and by means of social media which is a deviant of technology. Based on what has been claimed by Kabilan et al. (2010) learning communities can be built through collaborative work to boost the language. Moreover, social media is regarded as a tool through which the enhancement of such communities can be facilitated by urging communication and collaboration. Additionally, they cause the accomplishment of favorable learning consequences to be easier according to Yu et al. (2010). Consequently, as shown by Shoshani and Rose Braun (2007) learning collaboratively and creatively can be strengthened through social media and as a result of which engagement increases either in class or outside. Collaborative learning is concerned with interactions among students. Moreover, an opportunity has been provided by social media to boost their learning environment because only a part of the learning process occurs during the class; therefore, students’ involvement can be enhanced (Chen and Bryer, 2012; Friesen and Lowe, 2012; Wodzicki et al., 2012). It was further proposed by Fewkes and McCabe (2012) that educators’ responsibility is to find ways to integrate social media into their classrooms. Students can be encouraged by educators to use social media to boost a sense of creativity by motivating them to probe the content material in a new way. It can be taken as an example, social media helps students to create real, creative materials, using tools including blogs, YouTube, and podcasts (Frye et al., 2010; Lamb and Johnson, 2010). And students are more likely to make a balance between their individualism and their need to communicate with others through a cooperative learning environment that encourages engagement (Shoshani and Rose Braun, 2007; Garrett, 2011).

Using technology independently does not necessarily result in learning. Aguilar-Roca et al. (2012) reported that taking notes by hand helps students accomplish better test scores compared with the ones who utilize laptops for taking notes. In addition, knowing something about computers has a good impact on students’ learning when they use online ways of learning (Appel, 2012; Top, 2012). However, a rich environment can be provided by the Internet because students access different types of information for various levels, yet care should be taken in order not to feel baffled or distracted finding their own appropriate materials. Students who mainly take online courses allocate more time to utilizing tools and social media than students who mainly take face-to-face classes. Therefore, social media helps create an environment in which critical thinking, collaboration, and engagement can be enhanced by complementing student coursework with external sources (Carini et al., 2006; Mazman and Usluel, 2010).

The purpose of a study conducted by Rajendran and Yunus (2021) was to evaluate the international office services at the university level, considering the potential for improving English proficiency and integration into the American community for second/foreign language learners. These programs actively involve students in practicing English and consist of activities aiming at different aspects of life at an American university. The purpose was to find out how these activities have a positive impact on the students’ language and social learning experience. The results provide a complete picture including language and cross-cultural opportunities in international office services related to the personal and social growth of international students. Mastering all four language skills is a top priority for both ESL and EFL learners around the world. Apparently speaking skills remain the most challenging of all other skills many learners still struggle to speak well. The results of the review suggest that the application of *Mobile Assisted Language Learning*, MALL, extends the concept of constructivist theory, promotes a stress-free environment, supports situational learning, and provides ease of use which all facilitate the learning process and improve engagement. Further analysis shows that the common built-in features and mobile applications of mobile devices can be used as potential tools to help learners improve their speaking skills in the MALL environment.

According to another study conducted by Al-Khalidi and Khouni (2021), Social media technology has become an important part of all fields, especially in education and EFL/ESL education. It has had a profound impact on education and learning systems, leading to the emergence of learning communities supported by collective interaction and involvement. EFL students’ attitudes toward the usage of social media platforms in the context of learning and teaching were scrutinized in this quantitative study. The findings showed that the majority of participants strongly believed in the benefits of social media platforms to support educational purposes and improve English skills. A study by Derakhshan et al. (2021) was carried out during the coronavirus pandemic to see what the reasons behind students’ boredom are and some solutions were also put forward. It should be taken into account that boredom leads to less engagement and students do not feel energetic to be involved in the activities. It was reported that 10 types of activities caused boredom in students, among which two of them seem more significant, homework assignments that have a demanding nature and the ones that cannot actively engage the students such as passive listening and reading in class. Participants found it boring to hear the unattractive presentations of their classmates, in addition to the long and monotonous readings by teachers, such as previously prepared materials and PowerPoint slides. Teachers who were supposed to provide materials that were fun and engaging seemed to be just worried about providing access to content. In the mentioned study one of the solutions to remove boredom, which is one of the most important reasons behind not being engaged in the class, was that teachers can incorporate at least some principles of collaborative learning into their activities to lessen the effects of boredom and its devastating impacts. So social networks can act as a motivator to decrease boredom in classes. Encalada and Sarmiento (2019) indicated that students boosted their vocabulary, enhanced pronunciation accuracy and raised their confidence in their capability to speak English through self-recording videos which then could be shared through social media. According to Kirkgöz (2011), students’ vocabulary was expanded and their oral skills were also enhanced after they were given a speaking task to record a video. It was reported that a sense of achievement could be developed in students through video or vlog projects and a sense of engagement could be also enhanced (Gromik, 2012, 2015; Aldukhayel, 2019). It was also shown that literacy proficiency could be improved through producing videos as speaking projects (McKenney and Voogt, 2011; Yang and Wu, 2012), students’ social skills while communicating with peers were also enhanced (Park, 2019), and active learning was promoted (Anas, 2019). It was revealed by Encalada and Sarmiento (2019) that students enhanced their vocabulary, pronunciation, and accuracy and built their confidence in their capability to speak English through self-recording videos as a speaking homework assignment which then could be shared through social media which is also another emblem of engaging students through the class and outside the class.

It is proven by the above studies that social media acts as a facilitator for students’ engagement both inside and outside the class because it is accessible everywhere and students do not feel worried to learn something new even after their class time at home and whenever they feel like studying despite the fact that several years ago the only way to increase one’s knowledge of English was joining a face-to-face class.

### Students’ engagement

Engagement is regarded as an integral part of the process of learning and is a multifaceted conception. It is divided into various categories: Emotional involvement, for example, when one is highly enthusiastic about the activity and feels less anxious and bored. The second category of engagement is cognitive involvement in learning strategies and self-use restrictions. Agent participation as another category of engagement is a conscious effort for the learning experience to be enriched (Veiga et al., 2014; Hiver et al., 2021). Within the above categories, participation in behavioral learning processes is particularly important as it relates to the authentic perception of individual learning capabilities (Dörnyei, 2019).

Engagement can also be seen from two different perspectives, inside and outside. The former means the amount of time and diligence that is allocated to the process of learning. The latter has a variety of learning options and potential consequences such as persistence and satisfaction (Harper and Quaye, 2009). Engagement is recognized as a behavioral tool to motivate students, and as a result, with care, development can occur throughout the learning process (Jang et al., 2010). Active participation in L2 instruction needs to be increased to prevent destructive behavior and reduce the value of negative emotions such as fear, frustration, and boredom. Another term, “dissatisfaction,” can be considered important because it can be considered the opposite of engagement. Dissatisfaction is concerned with indifference, disgust, resignation, and reduced effort. Therefore, looking at boredom using the following factors can enhance the perception of boredom as a complex emotion and deal with it more systematically (Derakhshan et al., 2021). As mentioned earlier, students should have been taught to ask questions actively. This is a paramount part of thinking critically and is perceived as the core of student involvement. Students are highly likely to learn when they ask their questions. Some of the students are habituated to not being faced with the consequences of their irrational decisions because they have always needed someone to support them or maybe take the responsibility for their own mistakes (Rezaei et al., 2011).

Adaptive e-learning is seen as a stimulus to support learning and improve student involvement. Therefore, designing a suitable adaptive e-learning environment contributes to the personalization of teaching to enhance learning outcomes. The results of a study conducted by El-Sabagh (2021) show the possibility of an adaptive e-learning environment that motivates students to learn. According to Qureshi et al. (2021), the development of classrooms for active learning will be part of a broader educational initiative for students to engage in learning. To this end, we investigated the impact of social factors on collaborative learning and involvement that affect student learning outcomes. It was proposed that social factors, such as interaction with peers and teachers, social presence, and the use of social media, have a positive effect on active co-learning and student participation and influence learning achievement. The results also showed that as online learning became more common in education, it was concluded that overall collaborative learning and inclusion of social factors improve student learning activities. Therefore, its use in education and learning in higher education institutions should be encouraged as it will affect the improvement of students’ academic achievement.

Students’ engagement and success are highly affected by the quality of the relationship between teachers and students (Zhang, 2020). As an example, people may suffer from financial poverty, leading to their unemployment. Likewise, lack of motivation may contribute to boredom or dropping out of university which means that regular interactions between teachers and students and also the learning atmosphere are the key factors for students’ engagement (Derakhshan et al., 2021). Having a close student-teacher relationship may enhance socio-emotional improvement, contributing to being adapted and successful in EFL contexts (Pishghadam et al., 2019).

### The advantages and disadvantages of utilizing social media and its impact on students’ engagement, YouTube as an example

Social media can be used inside and outside the class and without a doubt, the number one reason it can be used is to boost EFL learners’ engagement and communication. If it were not for social media, it would be difficult to keep in touch with students outside the class. In the new era of the educational system, students have been encouraged to feel autonomous and take responsibility for their own learning and social media has enabled them to reach this goal since they can interact with their peers and teachers even outside of the class. However, the question is how social media causes students’ involvement. Content for example can be opted for and it can be discussed by students through social media. They can also search for further information if needed and add their own part to the content so as to make it complete. In this way, they can boost their communication skills which are highly emphasized when learning a new language (even though accuracy and fluency are of paramount importance) and they can partake actively in these discussions. The second important point that can be taken into consideration is that students can learn more from their peers through social media and they can also be motivated by them; therefore, they are more inclined to engage in the activities. On the other hand, it might be claimed that interacting through social media would increase the risk of making mistakes and not being revised. It might be true but fear of making mistakes should not prevent a person from being actively engaged in the activities since it is a significant part of the learning process and how one’s language skills could be enhanced if no mistakes would be made. In this respect, teachers should draw students’ attention to the point that they are supposed to be more careful about what their peers send and write through social media. Students should also be urged to self-correct their own mistakes or their peers’ mistakes if their knowledge of the language is good enough. Furthermore, teachers had better control the whole process to make it much more organized for the students. Students should not be left without any help even out of class because it may cause them to feel baffled or distracted. All this process should be regarded as a curriculum in order to be taken seriously by the students even though in many circumstances it has been considered an extracurricular activity because it is supposed to be done outside of class. Consequently, engagement is what should be urged among students and social media play a crucial role in it.

English learners in the context of EFL, where grammatical instruction is still prevalent, often lack the opportunity to use the target language both inside and outside the classroom. But when social media is considered, students are not limited to just learning through the class, but there is something beyond the class environment that allows learners to learn wherever and whenever they want. From another point of view, as a consequence of the COVID-19 pandemic, most learning around the world has been streamed online. Learners who were previously involved in traditional learning are now faced with the new challenge of a significant increase in e-learning. This dramatic change can affect their learning behavior and they do not resist the changes anymore. This can have a significant impact on their learning efforts. Before that, they have barely welcomed a change in the way they studied, but right after Covid 19 outbreak, they have been forced to use social media as a means of learning. Because through covid 19 pandemic everyone has lost his interest in learning a new language, social media could act as reinforcement so as to encourage students to be engaged in the learning process and enhance their knowledge of English even if there have been no face-to-face classes to join. The role of social media through the pandemic has been so tremendous that it cannot be denied throughout this period of time because students’ inactive English skills could be activated by communicating with other English learners all over the world. From some students’ point of view, learning just happens when eye contact is made and physical interaction is used, but little by little they come to the recognition that it would not be difficult to communicate through social media for learning and being taught a foreign language.

Another point that can be stressed is that social media with all its great items has also brought its own negative effects. It causes the students to have a sedentary lifestyle in which physical activity plays no role despite the fact that in Total Physical Response, being physically active has had a significant impact on learning. But when using social media, one has to be bound to his seat in order to feel focused and do what he is supposed to do and that is the reason why they may get used to this destructive way of lifestyle and lose their interest in face-to-face communication through the class since they have spent most their time interacting with people through the virtual world. Another highlighted point is that some students have been given the ability to use the virtual word as a way of learning; however, some other students are not enthusiastic about learning through social media since they think their health might be at risk, for instance, their eyesight might be weakened, or they typing speed would not be as good as their handwriting. Hence, they are thought of as weak students in spite of the fact that they are not necessarily weak students in the real world. Just their preference goes to interacting with other students in real face-to-face learning contexts rather than reaching them through social media.

It was confirmed by several studies that YouTube is one of the useful language learning teaching tools because it has different types of learning videos and is classified as a rich source of teaching materials for young bilingual students (Moghavvemi et al., 2018; Sakkir et al., 2020; Wang and Chen, 2020). A similar study indicated the impact of utilizing YouTube as supplementary material for listening comprehension and scrutinized students’ improvement after 5-week treatments conducted by Chien et al. (2020). It was claimed that YouTube was able to enhance the learning environment and build students’ motivation to be engaged and to learn English, especially in improving students’ listening comprehension. Chen and Chen (2021) also shared the same idea that YouTube videos could appeal to the students’ full attention which is one of the most important factors which help them be actively engaged in the class and make students perform well. The most crucial point was teachers’ need to keep an eye on choosing the right learning videos and control the proportion of the class activity to create good learning outcomes (Chien et al., 2020). Sari et al. (2020) explored the impact of YouTube videos on students’ creativity during the lesson. The values of YouTube videos can improve students’ creativity, interest, and motivation, and students were inspired to create new things, that is why they were more likely to be involved in the activity. Various educational videos, such as instructional videos, video tutorials, and animation videos, made students able to develop individual capabilities such as slime making, crafts, painting, and role-playing inspired by watching YouTube. Interactive activities helped students generate interesting ideas in the learning process. This was supported by the statement from Orús et al. (2016) that creativity is formed from new situations and concerns that modify original ideas with something new and unique. Besides improving the students’ vocabulary, listening skills, and creativity, the use of YouTube videos has also been observed in terms of the effects on students’ learning outcomes, satisfaction, and self-improvement. Students took part in the video project creation using YouTube. Students who were active in creating videos on YouTube were proven to affect their competence and build a good academic performance. Azer et al. (2018) stated that YouTube had positive contributions, especially in subjects they did not understand and students watched videos from YouTube as a substitute for textbooks. For example, instead of being taught some materials through the class, students can watch the lessons on YouTube and improve their English. It is where students’ engagement out of class can come to meaning.

## Recommendations for future research

Three groups can be benefited from this study. First teacher educators have the responsibility of training teachers. Teachers should be taught how to use social media to stretch students’ imaginations and boost their creativity. Without a doubt, there is a difference between students who have been actively engaged in social media to boost their knowledge of the language and a student never being given the opportunity to use social media as means of learning. The former group has been given the opportunity to share its content through the platforms through which many learners can learn a foreign/s language.

Another point that could be taken into account is that social media provides teacher educators and teachers themselves with customized ways of teaching. In this respect, students are highly likely to be engaged in the activities since the emphasis was put on individual differences and everyone can be given precise attention to use and sometimes produce his own content. While in the traditional way of teaching, students were provided with a specific type of task as their homework assignments and they were supposed to do it on their own. Instead with the help of social media, even if students are given the same task, they are expected to work on it in peer groups and in this way collaborative work is enhanced among students, and as a result of which their motivation for learning can be boosted and they can learn from their own mistakes without being directly reprimanded by their teachers.

On the other hand, students can be benefited from this way of learning and teaching since they can learn through group work and both inside and outside the class. In flipped learning, the attention was focused on learning not only in the classroom but also when students are out of the class. Because in the modern way of learning, the effort is made to make students feel autonomous and teach them how to learn, considering that social media can play a crucial role in it. Hence, more studies need to be conducted to go through the details of what can be done in social media outside the class so as to cause students to be more engaged and inspired to learn more and more and enjoy the learning process.

## Author contributions

All authors listed have made a substantial, direct, and intellectual contribution to the work and approved it for publication.

## Conflict of interest

The authors declare that the research was conducted in the absence of any commercial or financial relationships that could be construed as a potential conflict of interest.

## Publisher’s note

All claims expressed in this article are solely those of the authors and do not necessarily represent those of their affiliated organizations, or those of the publisher, the editors and the reviewers. Any product that may be evaluated in this article, or claim that may be made by its manufacturer, is not guaranteed or endorsed by the publisher.
